# Efficient Vision Mamba for MRI Super-Resolution via Hybrid Selective Scanning

**Published:** 2025-12-25

**Authors:** Mojtaba Safari, Shansong Wang, Vanessa L Wildman, Mingzhe Hu, Zach Eidex, Chih-Wei Chang, Erik H Middlebrooks, Richard L. J Qiu, Pretesh Patel, Ashesh B. Jani, Hui Mao, Zhen Tian, Xiaofeng Yang

**Affiliations:** 1 Department of Radiation Oncology and Winship Cancer Institute, Emory University, Atlanta, GA, United States; 2 Department of Radiology, Mayo Clinic, Jacksonville, FL, United States of America; 3 Department of Radiology and Imaging Science and Winship Cancer Institute, Emory University, Atlanta, GA, USA; 4 Department of Radiation and Cellular Oncology, University of Chicago, Chicago, IL, United States

**Keywords:** MRI, Deep learning, ultra high field MRI, super-resolution, state-space model, SSM

## Abstract

**Background.:**

High-resolution MRI is essential for accurate diagnosis and treatment planning, but its clinical acquisition is often constrained by long scanning times, which increase patient discomfort and reduce scanner throughput. While super-resolution (SR) techniques offer a post-acquisition solution to enhance resolution, existing deep learning approaches face trade-offs between reconstruction fidelity and computational efficiency, limiting their clinical applicability.

**Purpose.:**

This study aims to develop an efficient and accurate deep learning framework for MRI super-resolution that preserves fine anatomical detail while maintaining low computational overhead, enabling practical integration into clinical workflows.

**Materials and Methods.:**

We propose a novel SR framework based on multi-head selective state-space models (MHSSM) integrated with a lightweight channel multilayer perceptron (MLP). The model employs 2D patch extraction with hybrid scanning strategies (vertical, horizontal, and diagonal) to capture long-range dependencies while mitigating pixel forgetting. Each MambaFormer block combines MHSSM, depthwise convolutions, and gated channel mixing to balance local and global feature representation. The framework was trained and evaluated on two distinct datasets: 7T brain T1 MP2RAGE maps (142 subjects) and 1.5T prostate T2w MRI (334 subjects). Performance was compared against multiple baselines including Bicubic interpolation, GAN-based (CycleGAN, Pix2pix, SPSR), transformer-based (SwinIR), Mamba-based (MambaIR), and diffusion-based (I^2^SB, Res-SRDiff) methods.

**Results.:**

The proposed model demonstrated superior performance across all evaluation metrics while maintaining exceptional computational efficiency. On the 7T brain dataset, our method achieved the highest structural similarity (SSIM: 0.951 ± 0.021) and peak signal-to-noise ratio (PSNR: 26.90 ± 1.41 dB), along with the best perceptual quality scores (LPIPS: 0.076 ± 0.022; GMSD: 0.083 ± 0.017). These results represented statistically significant improvements over all baselines (*p <* 0.001), including a 2.1% SSIM gain over SPSR and a 2.4% PSNR improvement over Res-SRDiff. For the prostate dataset, the model similarly outperformed competing approaches, achieving SSIM of 0.770 ± 0.049, PSNR of 27.15 ± 2.19 dB, LPIPS of 0.190 ± 0.095, and GMSD of 0.087 ± 0.013. Notably, our framework accomplished these results with only 0.9 million parameters and 57 GFLOPs, representing reductions of 99.8% in parameters and 97.5% in computational operations compared to Res-SRDiff, while also substantially outperforming SwinIR and MambaIR in both accuracy and efficiency metrics.

**Conclusion.:**

The proposed framework provides a computationally efficient yet accurate solution for MRI super-resolution, delivering well-defined anatomical details and improved perceptual fidelity across anatomically distinct datasets. By significantly reducing computational demands while maintaining state-of-the-art performance, the model offers strong potential for clinical translation and scalable integration into future imaging workflows.

## 1 Introduction

Magnetic resonance imaging (MRI) is an essential used modality in both clinical and research settings due to its excellent soft-tissue contrast, high acquisition flexibility, and lack of ionizing radiation exposure. For example, quantitative 3D magnetization-prepared 2 rapid acquisition gradient echo (MP2RAGE) T1 mapping eliminates receptive field bias and first-order transmit field inhomogeneities in brain imaging. These maps enable precise diagnostic and treatment planning by Identifying hypoxic regions that can guide adaptive dose-painting radiotherapy [1, 2]. Likewise, anatomical T2-weighted (T2w) MRI provides superior soft-tissue contrast, making it indispensable for prostate cancer detection, staging, treatment planning, and longitudinal surveillance [3]. These examples underscore the importance of achieving high-resolution MRI across diverse anatomical sites and clinical tasks.

However, MRI resolution is fundamentally limited by hardware constraints and the extended acquisition times required for fine spatial detail. Higher resolution demands longer scans, which increase patient discomfort, heighten the risk of motion artifacts, and reduce scanner throughput [4]. Furthermore, high-resolution imaging typically requires high-field strength scanners (3T and above), which remain costly and inaccessible to many healthcare settings. To address these limitations, deep learning (DL)-based reconstruction methods have achieved impressive results, but typically requiring access to raw *k*-space data, under-sampling masks, and coil sensitivity maps [5]. In practice, access to raw *k*-space data might be limited by multiple factors such as vendor restrictions, proprietary data formats, and institutional privacy regulations. In contrast, DL-based super-resolution (SR) approaches attempt to recover high-resolution (HR) images from low-resolution (LR) inputs without such priors, offering a more flexible and widely applicable solution [6].

Convolutional neural network (CNN)-based SR models have shown promising results in medical imaging [7, 8], yet their inherently limited receptive field restricts the ability to capture long-range dependencies. These dependencies refer to spatial relationships between distant regions of an image that are important for preserving global structural and contextual consistency [9]. Vision Transformers (ViTs) address this limitation through a multi-head self-attention mechanism, which allows each image patch to interact with all other patches in the feature space [10, 11]. However, the quadratic computational complexity of self-attention with respect to input size makes ViTs computationally expensive for high-resolution MRI, where large image dimensions dramatically increase memory and processing demands. To improve efficiency, variants such as SwinIR [12] and Uformer [13] adopt window-based attention, which confines the attention operation to local non-overlapping regions instead of the entire image, thereby reducing complexity while retaining local structural context. Building on this concept, MB-TaylorFormer [14] further approximates multi-head self-attention using a Taylor-series expansion to achieve a better trade-off between accuracy and efficiency. Nevertheless, despite these advancements, the balance between high performance and computational efficiency remains a key challenge [15], as achieving global context modeling with reduced attention complexity often leads to performance degradation or limited scalability [16].

To further address computational efficiency, SwinIR employs a Swin Transformer backbone with shifted windows to balance local and global feature interactions while reducing memory overhead [17]. More recently, MambaIR introduced a vision Mamba architecture for image restoration, utilizing state-space models with selective scanning to achieve linear-time complexity [18]. However, these methods still face challenges in preserving fine anatomical details while maintaining computational efficiency across diverse MRI contrasts and anatomical regions.

Recently, state space models (SSMs) have emerged as efficient alternatives for sequential modeling. The Mamba framework [19] introduced selective state spaces that achieve linear-time complexity with respect to input length, enabling efficient processing of long sequences without the quadratic computational cost of self-attention. Unlike transformers, which explicitly compute pairwise interactions between all tokens, Mamba leverages a recurrent state update mechanism that implicitly captures long-range dependencies through selective gating. This design substantially reduces memory usage and computational overhead while maintaining the ability to model global context. Building on this foundation, vision Mamba variants have been adapted for image restoration and MRI reconstruction [20, 21, 22]. Similar to ViTs, vision Mambas represent images as sequences of patches but employ selective two-dimensional scanning strategies to further improve computational efficiency [19]. However, existing implementations can be susceptible to pixel forgetting when forming horizontal and vertical tokens [23], and densely sampled input patches may impose additional computational overhead.

In this study, we propose an efficient Vision Mamba framework for MRI super-resolution. Our contributions are three-fold: (i) a hybrid selective scanning strategy (vertical, horizontal, diagonal) to mitigate pixel forgetting and enhance long-range dependency modeling, (ii) integration of a lightweight channel MLP to reduce parameter overhead while preserving representational power, and (iii) application and validation on two distinct datasets: 7T MP2RAGE brain T1 maps and 1.5T prostate T2w images. By combining efficiency with high-fidelity reconstruction, the proposed framework provides a solution to bridge the gap between research-oriented SR models and clinically scalable MRI applications.

## 2 Materials and methods

### 2.1 Vision Mamba

**Figure 1:**
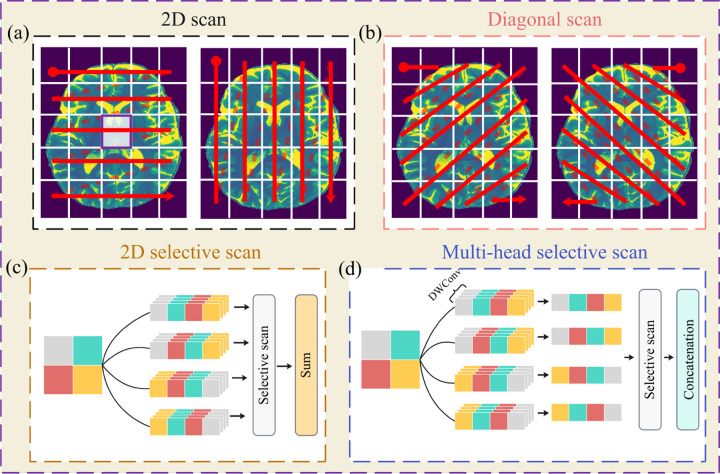
Selective scanning strategies in Vision Mamba. (a) Horizontal and vertical scanning may separate central pixels from their diagonal neighbors. (b) Diagonal scanning preserves spatial adjacency. (c) Dense extraction increases parameter overhead. (d) Our efficient variant uses depthwise convolutions for preprocessing.

Similar to the ViT, the Vision Mamba architecture partitions an input image into non-overlapping 2D patches, which are then processed through a SSM using selective scanning strategies (Figures 1(a)–(b)). Conventional approaches adopt horizontal and vertical scans [24, 25], but these separate central pixels (white box in Figure 1(a)) from their diagonal neighbors, thereby limiting the ability of Vision Mamba models to capture long-range spatial dependencies. In contrast, diagonal scanning preserves adjacency between central and diagonal pixels (Figure 1(b)) [26, 27]. In this study, we employ a hybrid strategy that combines vertical, horizontal, and diagonal scans to form the input sequences.

Densely extracted patches require separate sets of parameters, which can increase computational cost of selective scanning (Figure 1(c)). To address this, our efficient variant first applies depthwise convolutional layers to preprocess patches, reducing the number of trainable parameters while maintaining representational capacity. The outputs are concatenated to form the input sequence to the SSM (Figure 1(d)).

**Figure 2:**
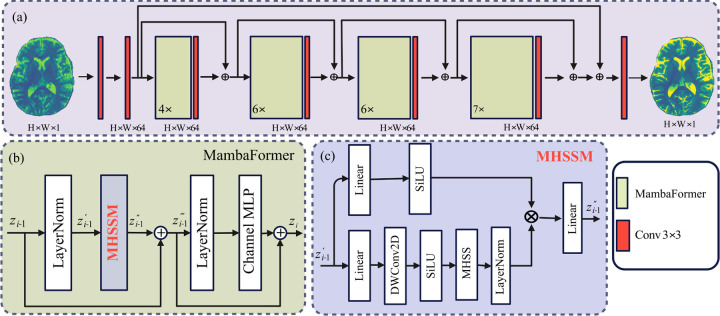
Proposed Vision Mamba framework for MRI super-resolution. (a) Hybrid selective scanning extracts patch sequences. (b) Each MambaFormer block integrates LN, MHSSM, and Channel MLP with residual connections. (c) Multi-head selective state-space modeling runs m parallel scans with adaptive step sizes.

The overall framework for reconstructing a HR image **x**^**HR**^ from a LR input **x**^**LR**^ is shown in Figure 2. The architecture consists of four MambaFormer stages, repeated 4, 6, 6, and 7 times, respectively (Figure 2(a)). Residual connections are employed throughout to stabilize training. Each block integrates Layer Normalization (LN), a Multi-Head SSM Mamba (MHSSM) module, and a Channel Multi-Layer Perceptron (MLP), with residual connections applied after both sub-layers (Figure 2(b)):

(1) $$z_{i-1}^{\prime \prime \prime }=z_{i-1}+\text{MHSSM}\left(\text{LN}\left(z_{i-1}\right)\right),\;\;z_{i}=z_{i-1}^{\prime \prime \prime }+\text{Channel,}\;\text{MLP}\left(\text{LN}\left(z_{i-1}^{\prime \prime \prime }\right)\right),$$

where $z_{i-1}$ and $z_{i}$ are the input and output features of the i-th block, respectively.

The Channel MLP is designed to be lightweight while preserving expressivity, inspired by recent efficient vision architectures [28, 29]. Given an input $x\in {\mathbb{R}}^{B\times C\times H\times W}$, a 1 × 1 convolution expands the channel dimension by a factor *α* (set to *α* = 2). The features are then split into two halves $\left(x_{1,\;}x_{2}\right)$, and a multiplicative gating computes $\left(x_{1}⊙x_{2}\right)$. A second 1 × 1 convolution projects the result back to the original dimension:

(2) $$\text{Channel}\;\text{MLP}\left(x\right)=W_{\text{out}}\left({\left(W_{\text{in}}x\right)}_{1:\frac{C\alpha }{2}}⊙{\left(W_{\text{in}}x\right)}_{\frac{C\alpha }{2}:C\alpha }\right),$$

where $W_{\text{in}}$, $W_{\text{out}}$ denote learned pointwise (1×1) convolutions. This design preserves the expressivity of a two-layer MLP, but is parameter-efficient.

The MHSSM module shown in Figure 2(c) begins with a linear projection, followed by a depthwise 3 × 3 (DWConv2D) convolution and SiLU activation for local feature mixing. The processed features are then passed into a multi-head selective state-space (MHSS) layer, which runs m parallel recurrent scans with adaptive step sizes. For head i, the update rule is:

(3) $$h_{t}^{\left(i\right)}=e^{\Delta_{t}^{\left(i\right)}A\left(i\right)}\;h_{t-1}^{\left(i\right)}+\Phi \left(\Delta_{t}^{\left(i\right)},A^{\left(i\right)}\right)B^{\left(i\right)}u_{t},\;y_{t}^{\left(i\right)}=C^{\left(i\right)}h_{t}^{\left(i\right)}+D^{\left(i\right)}u_{t},$$

where $A^{\left(i\right)}=\text{diage}\left(-e^{a^{\left(i\right)}}\right)$ ensures stability, and $\Phi \left(\Delta ,A\right)=\left(e^{\Delta A}-I\right)A^{-1}$ [19, 30]. The step sizes $\Delta_{t}^{\left(i\right)}$ are predicted by a lightweight channel MLP with a softplus reparameterization. Outputs from all heads are merged, layer-normalized, optionally gated, and projected back to the model dimension by a linear layer.

Finally, a residual connection between the input features and output features was used to stabilize the training process before projecting the features into the image channel using a 3 × 3 convolution layer. The reconstructed high-resolution image ${\hat{x}}^{HR}$ was estimated by minimizing $\ell_{1}$ and learned perceptual image patch similarity (LPIPS) loss $\ell_{p}$. The $\ell_{1}$ loss is able to generate images with better

(4) $$𝓛=\lambda ∥{\hat{x}}^{HR}-x^{HR}{∥}_{1}^{1}+\ell_{p}\left({\hat{x}}^{HR},x^{HR}\right)$$

where λ=4 was used to put more weight on $\ell_{1}$ loss than the $\ell_{p}$.

### 2.2 Quantitative and statistical analysis

We compared our method against six benchmark approaches: Bicubic interpolation, Pix2pix [31], CycleGAN [32], SPSR [33], I^2^SB, SwinIR, MambaIR, and Res-SRDiff [34]. All comparative models were trained using their original hyperparameters.

The quality of the reconstructed HR image ${\hat{x}}^{HR}$ was evaluated using four quantitative metrics: peak signal-to-noise ratio (PSNR), structural similarity index (SSIM) [35], gradient magnitude similarity deviation (GMSD) [36], and learned perceptual image patch similarity (LPIPS) [37]. PSNR measures residual error between the reconstructed and ground-truth images; its logarithmic scale better reflects human visual perception [38], with higher values indicating better reconstruction. SSIM, GMSD, and LPIPS assess structural similarity and perceptual quality: higher SSIM (range –1 to 1, typically close to 1 in practice) indicates better agreement, while lower GMSD and LPIPS correspond to improved perceptual fidelity.

For each metric–method combination, descriptive statistics (mean and standard deviation) were reported. To assess overall group differences, we employed the non-parametric Kruskal-Wallis omnibus test separately for each metric. When omnibus significance was detected, post-hoc pairwise comparisons were conducted using Dunn’s test with Holm correction for multiple comparisons. All statistical analyses were performed in R (version 4.3), with significance defined as *p <* 0.05.

These metrics were selected to capture both fidelity (PSNR, SSIM) and perceptual quality (GMSD, LPIPS), thereby providing a comprehensive evaluation of reconstruction performance. Non-parametric tests were employed because distributional assumptions of normality were not satisfied across test samples (Shapiro-Wilk test, *p <* 0.05), ensuring robustness of the statistical comparisons.

### 2.3 Patient data acquisition and preprocessing

Two datasets were employed for training and evaluation: (i) an institutional ultra-high-field 7T brain T1 MP2RAGE dataset [39], and (ii) the publicly available ProstateX axial T2w prostate cancer dataset [40]. These datasets represent distinct anatomical regions and contrasts, enabling comprehensive evaluation of the proposed framework across neuroimaging and oncologic applications.

The institutional brain cohort consisted of 142 patients with confirmed multiple sclerosis. Data were retrospectively collected under Mayo Clinic IRB approval, with anonymization performed in accordance with institutional policies. The dataset was split into non-overlapping subsets for training (121 patients, 14,566 axial slices) and testing (21 patients, 2,552 slices). Imaging was performed on a 7T Siemens MAGNETOM Terra system equipped with an 8-channel transmit/32-channel receive head coil. Acquisition parameters were: TR = 4.5 s, TE = 2.2 ms, TI_1_/TI_2_ = 0.95/2.5 s, FA_1_/FA_2_ = 6°/4°, field-of-view (FOV) = 230 × 230 mm^2^, matrix size = 288 × 288, and isotropic resolution of 0.8 × 0.8 × 0.8 mm^3^, with a total scan time of 8:44 minutes. Brain masks were generated from inversion-1 images using FSL BET [41] and applied to remove extracranial signal from the T1 MP2RAGE maps. For model input, T1 maps were down-sampled by a factor of 4 in each spatial dimension, yielding a voxel size of 3.2 × 3.2 × 3.2 mm^3^.

From the ProstateX dataset, 334 patients were randomly selected. These were partitioned into training (268 patients, 10,480 slices) and evaluation (66 patients, 2,668 slices) sets. Data were acquired on a 1.5T Siemens scanner with imaging parameters: TR = 2.2 s, TE = 202 ms, FA = 110°, matrix size = 256 × 256, and voxel size of 0.66 × 0.66 × 1.5 mm^3^. T2w images were down-sampled by factors of 9 in-plane and 2 through-plane, resulting in an effective voxel size of 2 × 2 × 3 mm^3^.

Down-sampling of both the 7T brain T1 maps and the prostate T2w images was performed in the image domain using the SimpleITK.Resample package (version 2.1.1) [42], with linear interpolation.

## 3 Results

### 3.1 Brain T1 maps

**Figure 3:**
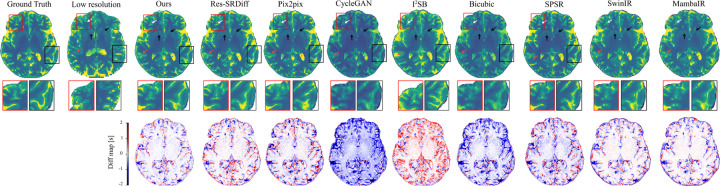
Qualitative comparison of super-resolution methods on 7T brain T1 MP2RAGE maps. The first row shows reconstructed images, the second row is the zoomed-in regions, and the last row depicts difference maps with respect to the ground-truth HR image. Arrows highlight cortical and subcortical structures (white: cortical ribbon; black: caudate nucleus and putamen; red: subtle tissue boundaries).

Figure 3 shows qualitative comparisons of super-resolution results on 7T brain T1 MP2RAGE maps. The first row displays reconstructed images, while the second row depicts difference maps relative to the ground-truth HR image. The proposed method yields the most faithful reconstruction of fine anatomical details. Subtle cortical and subcortical structures (white arrows) are more sharply delineated, and the head of the caudate nucleus and putamen (black arrows) are more accurately recovered compared to competing approaches. Both our model and Res-SRDiff demonstrate improved recovery of these regions, whereas Bicubic and CycleGAN produce pronounced blurring and loss of structural definition. Pix2pix and SPSR achieve moderate improvements but still fail to preserve fine tissue boundaries. SwinIR shows reasonable global consistency but exhibits residual blurring in cortical ribbon regions, while MambaIR captures local edge details but introduces minor inconsistencies in subcortical structures. The difference maps further support these observations: our method yields the lowest overall error relative to the ground truth, followed closely by Res-SRDiff. In contrast, Bicubic, CycleGAN, SwinIR, and MambaIR exhibit higher residual variations, reflecting suboptimal structural fidelity. Collectively, these qualitative results highlight the capability of our approach to preserve anatomically relevant features while minimizing reconstruction artifacts.

**Figure 4:**
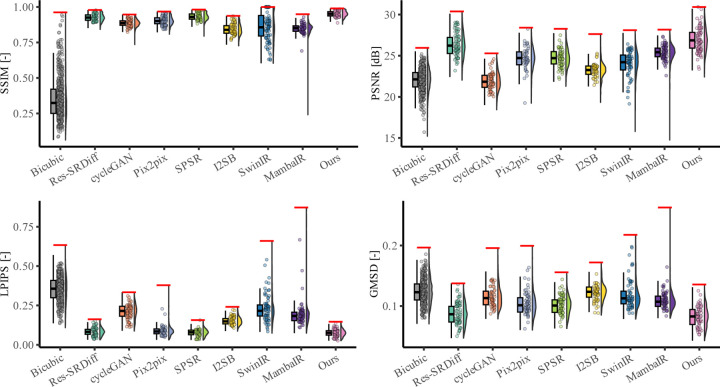
Quantitative evaluation of super-resolution methods on 7T brain T1 MP2RAGE maps. Bar plots show mean ± standard deviation of SSIM, PSNR, LPIPS, and GMSD across the test cohort. Higher SSIM/PSNR and lower LPIPS/GMSD indicate better performance.

Quantitative evaluation across four image quality metrics (SSIM, PSNR, LPIPS, and GMSD) further demonstrates the superiority of the proposed method (Table 1, Figure 4). Our model achieved the highest mean SSIM score (0.951 ± 0.021), surpassing all competing techniques, with SPSR ranking second (0.932 ± 0.025). For PSNR, our model reached 26.900 ± 1.410 dB, outperforming Res-SRDiff (26.282 ± 1.418 dB) and substantially exceeding Pix2pix, SPSR (∼24.7 dB), SwinIR (24.022 ± 1.615 dB), and MambaIR (25.309 ± 1.086 dB). In terms of perceptual similarity (LPIPS), our method obtained the lowest score (0.076 ± 0.022), indicating superior perceptual fidelity compared with SPSR (0.078 ± 0.021), Res-SRDiff (0.083 ± 0.024), SwinIR (0.229 ± 0.079), and MambaIR (0.188 ± 0.071). Finally, for distortion sensitivity (GMSD), our approach again delivered the best value (0.083 ± 0.017), outperforming Res-SRDiff (0.086 ± 0.017), SwinIR (0.117 ± 0.022), MambaIR (0.110 ± 0.019), and all other baselines.

Statistical testing confirmed the robustness of these findings. The Kruskal-Wallis omnibus test revealed significant group differences for all four metrics (SSIM: *p <* 0.001; PSNR: *p <* 0.001; LPIPS: *p <* 0.001; GMSD: *p <* 0.001). Post-hoc Dunn tests with Holm-adjusted *p*-values showed that our method significantly outperformed nearly all baselines across all metrics (adjusted *p <* 0.001 in most pairwise comparisons). These results confirm that the proposed framework consistently achieves statistically significant improvements in both fidelity- and perceptual-based metrics, establishing its robustness compared to the baseline models.

Importantly, these gains were achieved with substantially improved computational efficiency. Our model requires only 0.9M parameters and 57 GFLOPs, compared with Res-SRDiff (394M parameters, 2316 GFLOPs), SPSR (96M parameters, 871 GFLOPs), SwinIR (2M parameters, 369 GFLOPs), and MambaIR (1M parameters, 113 GFLOPs). This exceptional reduction highlights the favorable trade-off between accuracy and computational cost, making the method highly suitable for scalable clinical integration. Results are reported with two decimal places in tables for readability and three decimals in the text to emphasize fine-grained differences.

### 3.2 Pelvic T2w images

**Figure 5:**
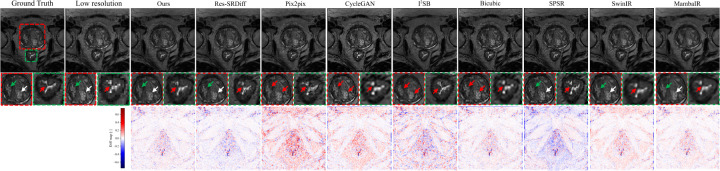
Visual comparison of super-resolution approaches on a representative axial prostate T2w MRI slice. Top panels display the ground truth image together with outputs from competing reconstruction methods. The second row provides magnified views from prostate and bladder regions of interest, chosen to emphasize structural detail. Colored arrows indicate specific aspects under evaluation: green arrows indicate edge delineation of fine tissue, white arrows indicate continuity of anatomical features, and red arrows indicate areas sensitive to artifacts. The bottom panels show voxel-wise difference maps relative to the ground truth.

Figure 5 presents a qualitative comparison of super-resolution results on axial T2w pelvic MRI. The ground-truth HR image (leftmost) clearly delineates anatomical boundaries, including the prostate capsule (green arrow), lesion region (white arrow), and surrounding structures (red arrow). Bicubic interpolation fails to recover fine anatomical detail, producing blurred boundaries and oversmoothed textures. GAN-based approaches such as CycleGAN and Pix2pix partially restore high-frequency components but introduce hallucinated structures and amplified noise, as seen in irregular residual patterns. SPSR demonstrates improved texture recovery, although boundary sharpness remains suboptimal. Transformer-based SwinIR shows reasonable global consistency but exhibits edge oversmoothing in fine tissue boundaries, while Mamba-based MambaIR captures local details but occasionally introduces minor artifacts in homogeneous regions. Diffusion-based methods (I^2^SB and Res-SRDiff) achieve higher fidelity, yet residual artifacts and structural inconsistencies are still visible in lesion-adjacent regions.

In contrast, the proposed method reconstructs sharper anatomical edges and preserves tissue continuity, closely matching the ground-truth HR reference. Lesion boundaries and the prostate capsule are more accurately delineated, and residual maps confirm lower reconstruction error relative to competing approaches. These qualitative findings align with the quantitative results summarized in Table 1, demonstrating that our model not only enhances perceptual similarity but also reduces distortion and preserves diagnostically relevant structures.

**Figure 6:**
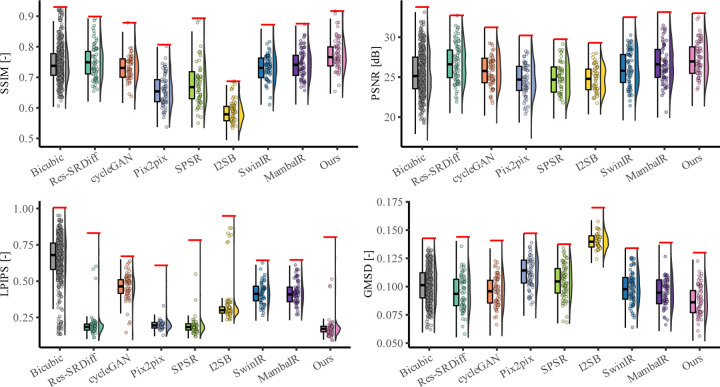
Quantitative comparison of super-resolution models on the prostate (axial T2w MRI) dataset. Bar plots show average performance across four metrics: SSIM (higher is better), PSNR (higher is better), LPIPS (lower is better), and GMSD (lower is better).

Quantitative comparisons on the prostate dataset are summarized in Table 1 and illustrated in Figure 6. Across all four evaluation metrics, our model consistently outperformed existing approaches. Specifically, it achieved the highest SSIM (0.770 ± 0.049) and PSNR (27.15 ± 2.19 dB), while also obtaining the lowest LPIPS (0.184 ± 0.096) and GMSD (0.083 ± 0.013). These results confirm that the proposed method improves both structural fidelity and perceptual quality compared with the baselines.

Figure 6 further illustrates these findings: Bicubic interpolation exhibits the lowest SSIM and PSNR, while GAN-based models (CycleGAN, Pix2pix, SPSR) show moderate improvements but remain inferior in perceptual similarity (higher LPIPS). Transformer-based SwinIR and Mamba-based MambaIR demonstrate intermediate performance, with SwinIR achieving better structural metrics but higher perceptual errors, and MambaIR showing competitive GMSD but suboptimal SSIM. Diffusion-based methods (Res-SRDiff, I^2^SB) achieve higher PSNR but still underperform compared to our model in perceptual quality and distortion sensitivity. Our approach consistently achieved the best performance across all metrics, with clear margins over both GAN-, transformer-, Mamba-, and diffusion-based baselines.

To confirm statistical significance, we conducted Kruskal-Wallis omnibus tests, which revealed significant overall differences across all metrics (*p <* 0.001). Post-hoc Dunn tests with Holm correction verified that our method significantly outperformed the comparative models across all four metrics (*p <* 0.001), with no statistically significant difference compared to Res-SRDiff on LPIPS. Together, these findings demonstrate that our method provides both statistically and practically significant improvements for prostate MRI super-resolution.

Importantly, these performance gains were achieved with substantially improved computational efficiency. As shown in Table 1, our method requires only 57 GFLOPs and 0.9M parameters, compared to 2316 GFLOPs and 394M parameters for Res-SRDiff and 871 GFLOPs with 96M parameters for SPSR. This demonstrates that the proposed model achieves state-of-the-art image quality at a fraction of the computational and memory cost, advantages that render it well suited for clinical deployment and integration.

## 4 Discussion

MRI remains one of the most versatile imaging modalities in both clinical and research applications, offering exceptional soft-tissue contrast and flexible imaging capabilities without exposing patients to ionizing radiation. However, its inherently lengthy acquisition times reduce scanner throughput and increase the likelihood of patient motion, leading to artifacts. This trade-off between spatial resolution and acquisition efficiency often necessitates compromises: increasing voxel size can shorten scan duration but reduces resolution and image sharpness, with diagnostic performance further degraded by partial volume effects [43].

In this study, we developed an efficient Vision Mamba model to reconstruct HR images from LR inputs. By leveraging hybrid scanning directions, our approach mitigates voxel forgetting (as illustrated in Figure 1(a)–(b)), while the multi-head selective state-space module (MHSSM; Figure 2(c)–(d)) captures both local and long-range dependencies. Despite requiring fewer than one million parameters, our study achieves competitive or superior performance compared with much larger models, including transformer-based SwinIR and Mamba-based MambaIR.

Our findings demonstrate that the model consistently delivers high-quality reconstructions across both brain and prostate MRI, while maintaining remarkable computational efficiency. In brain MRI, the method excelled at recovering fine cortical and subcortical structures, including the caudate nucleus and putamen, which are critical for neuroimaging analyses. As shown in Figure 3, subtle tissue boundaries were more sharply delineated compared with competing methods. Bicubic interpolation and CycleGAN exhibited substantial structural loss, while Pix2pix and SPSR partially restored anatomical features but failed to preserve finer details. Transformer-based SwinIR showed reasonable global consistency but suffered from edge blurring, and Mamba-based MambaIR captured local details but introduced inconsistencies in complex anatomical regions. Although Res-SRDiff provided competitive recovery, its perceptual fidelity remained inferior to our model.

Quantitative evaluation further supported these observations. The proposed method achieved the highest SSIM (0.951 ± 0.021) and PSNR (26.900 ± 1.410 dB) on the 7T brain dataset, while also recording the lowest LPIPS (0.076 ± 0.022) and GMSD (0.083 ± 0.017). Statistical testing confirmed significant differences across methods (*p <* 0.001 for all metrics), with Dunn post-hoc tests verifying that our model outperformed all baselines, including SwinIR and MambaIR. Importantly, these improvements were achieved with only 57 GFLOPs and 0.9M parameters, compared to Res-SRDiff (2316 GFLOPs, 394M parameters), SPSR (871 GFLOPs, 96M parameters), SwinIR (369 GFLOPs, 2M parameters), and MambaIR (113 GFLOPs, 1M parameters). These results suggest that architectural efficiency, rather than brute computational scale, can yield clinically meaningful advances.

A similar trend was observed in prostate MRI super-resolution. As shown in Figure 5, our method reconstructed sharper prostate capsule boundaries and more faithfully preserved lesion morphology compared with competing models. GAN-based approaches partially restored textures but often introduced hallucinations or amplified noise, limiting diagnostic reliability. Transformer-based SwinIR maintained global consistency but oversmoothed fine tissue boundaries, while Mamba-based MambaIR captured local edge details but occasionally produced artifacts in homogeneous regions. Diffusion-based methods achieved higher fidelity than GANs, but residual artifacts were still evident, especially near lesion-adjacent regions. In contrast, our approach minimized reconstruction error and preserved clinically relevant structures.

The quantitative results corroborated these findings: our method achieved the best SSIM (0.770 ± 0.049) and PSNR (27.15 ± 2.19 dB), while also recording the lowest LPIPS (0.184 ± 0.096) and GMSD (0.083 ± 0.013). These values significantly outperformed Bicubic, GAN-based, transformer-based (SwinIR), Mamba-based (MambaIR), and diffusion-based methods (*p <* 0.001 for all metrics), with only marginal differences relative to Res-SRDiff on LPIPS. Consistent with results of the brain dataset, the improvements were realized with substantially lower computational costs, underscoring the method’s suitability for large-scale or real-time deployment.

Taken together, the consistent superiority of our method across two anatomically and contrast-wise distinct datasets demonstrates both its robustness and generalizability. By combining high fidelity, perceptual accuracy, and lightweight design, the proposed model offers a practical step toward integrating super-resolution into clinical MRI workflows, where accuracy must be balanced with efficiency.

This study has several limitations that should be acknowledged. First, although both the brain and prostate datasets consist of full 3D MRI volumes, our proposed framework is implemented as a 2D slice-based super-resolution approach. This design leverages the efficiency of 2D convolutional and state-space modules, reducing computational complexity and enabling faster inference. However, the independent processing of slices does not enforce volumetric continuity, and thus subtle inconsistencies may arise across adjacent slices. Such discontinuities may limit downstream applications that rely on smooth 3D reconstructions, such as volumetric segmentation or radiomics analysis. Future work will investigate extending the framework to 3D architectures or introducing inter-slice regularization to ensure volumetric coherence.

Second, although our method demonstrated significant gains in both fidelity- and perceptual-based metrics, the evaluation was limited to two datasets with specific contrasts (7T T1 MP2RAGE brain maps and 1.5T axial T2w prostate images). While these datasets cover distinct anatomical regions, broader validation across additional organs, imaging modalities, and scanner vendors is needed to establish full generalizability. In line with recent work on foundation models in medical imaging, which emphasize the importance of large-scale, diverse training and evaluation for robust performance across tasks, extending our framework to more heterogeneous datasets will be a critical next step [44, 45].

Finally, despite the clear improvements in structural fidelity and perceptual similarity, we note that super-resolution can, in rare cases, introduce subtle alterations to fine anatomical structures, as shown in Figure 3 red arrows. While our residual analyses suggest lower error rates compared to competing models, such artifacts may still carry clinical consequences. Future directions should include uncertainty quantification and structure-preserving constraints to safeguard against the inadvertent modification of clinically relevant features.

## 5 Conclusion

This study presented a state space-driven framework for efficient and effective MRI super-resolution from low-resolution inputs. The proposed method integrates multihead selective state space modules with a lightweight channel design, enhancing computational efficiency compared with conventional transformer-based models, including SwinIR and MambaIR. Experimental results demonstrate that the framework can generate high-quality, high-resolution MRI images within clinically practical time frames. Owing to its reduced computational cost and scalability, the proposed approach holds strong potential for translation and deployment across diverse clinical imaging environments.

## Figures and Tables

**Table 1:** Quantitative results of super-resolution experiments on pelvic T2w MRI and 7T brain T1 MP2RAGE datasets, presented with metrics as rows and methods as columns. Values are mean±SD. The best scores for each metric are highlighted in bold, while the second-best are underlined. Arrows indicate the preferred direction.

Dataset	Metric	Bicubic	CycleGAN	Pix2pix	SPSR	SwinIR	MambaIR	I^2^SB	Res-SRDiff	Ours
Pelvic T2w MRI	PSNR [dB] ↑	25.47_±2.61_	25.84_±1.96_	24.83_±2.09_	24.74_±1.96_	26.06_±2.25_	0.74_±0.05_	26.78_±2.35_	**27.72**_±**2.26**_	27.14_±2.19_
	SSIM [-] ↑	0.75_±0.06_	0.73_±0.05_	0.66_±0.05_	0.68_±0.07_	0.73_±0.05_	0.74_±0.05_	0.70_±0.04_	0.75_±0.05_	**0.77**_±**0.05**_
	GMSD [-] ↓	0.10_±0.02_	0.10_±0.01_	0.11_±0.01_	0.11_±0.01_	0.10_±0.01_	0.09_±0.01_	0.14_±0.01_	**0.08**_±**0.02**_	**0.08**_±**0.01**_
	LPIPS [-] ↓	0.69_±0.15_	0.45_±0.10_	0.20_±0.05_	0.20_±0.09_	0.42_±0.08_	0.41_±0.08_	0.33_±0.13_	0.21_±0.11_	**0.18**_±**0.10**_
7T brain T1 MP2RAGE	PSNR [dB] ↑	22.00_±1.37_	21.89_±1.09_	24.63_±1.32_	24.76_±1.12_	24.02_±1.62_	25.31_±1.09_	23.22_±0.98_	26.28_±1.41_	**26.90**_±**1.41**_
	SSIM [-] ↑	0.31_±0.16_	0.86_±0.02_	0.90_±0.03_	0.93_±0.02_	0.86_±0.10_	0.85_±0.04_	0.84_±0.04_	0.92_±0.03_	**0.95**_±**0.02**_
	GMSD [-] ↓	0.12_±0.02_	0.12_±0.02_	0.10_±0.02_	0.10_±0.01_	0.12_±0.02_	0.11_±0.02_	0.12_±0.01_	**0.07**_±0.02_	**0.07**_±**0.02**_
	LPIPS [-] ↓	0.38_±0.07_	0.21_±0.05_	0.09_±0.04_	**0.08**_±0.02_	0.23_±0.08_	0.19_±0.07_	0.15_±0.03_	**0.08**_±**0.02**_	**0.08**_±**0.02**_
Model size	Flops [G]	x	18	9	871	369	113	497	1316	57
	Params [m]	x	89	57	96	2	1	114	394	0.9

## Data Availability

The ProstateX dataset is openly accessible through the TCIA portal (https://www.cancerimagingarchive.net/analysis-result/prostatex-seg-hires/). The institutional dataset used in this study contains sensitive patient information and therefore cannot be released publicly at the time of publication.
